# Chondrogenesis of Peripheral Blood-Derived Mesenchymal Stromal Cells

**DOI:** 10.3390/cells15050476

**Published:** 2026-03-06

**Authors:** Harish V. K. Ratna, Madhan Jeyaraman, Naveen Jeyaraman, Arulkumar Nallakumarasamy, Luise Schäfer, Filippo Migliorini, Sathish Muthu

**Affiliations:** 1 Department of Orthopaedics, ACS Medical College and Hospital, Dr MGR Educational and Research Institute, Chennai 600077, Tamil Nadu, India; harivk07@gmail.com (H.V.K.R.); madhanjeyaraman@gmail.com (M.J.); naveenjeyaraman@yahoo.com (N.J.); 2 Department of Regenerative Medicine, Agathisha Institute of Stemcell and Regenerative Medicine (AISRM), Chennai 600030, Tamil Nadu, India; arulmmcian@gmail.com (A.N.); drsathishmuthu@gmail.com (S.M.); 3 Department of Orthopaedics, Orthopaedic Research Group, Coimbatore 641045, Tamil Nadu, India; 4 Department of Orthopaedics, Jawaharlal Institute of Postgraduate Medical Education and Research (JIPMER), Karaikal 609602, Puducherry, India; 5 Department of Orthopaedic and Trauma Surgery, Eifelklinik St. Brigida, Kammerbruchstr. 8, 52152 Simmerath, Germany; luise.schaefer@artemed.de; 6 Department of Trauma and Reconstructive Surgery, University Hospital of Halle, Martin-Luther University Halle-Wittenberg, 06097 Halle (Saale), Germany; 7 Department of Life Sciences, Health, and Health Professions, Link Campus University, Via del Casale di San Pio V, 00165 Rome, Italy; 8 Central Research Laboratory, Meenakshi Medical College Hospital and Research Institute, Meenakshi Academy of Higher Education and Research, Chennai 631552, Tamil Nadu, India

**Keywords:** peripheral blood, mesenchymal stromal cells, chondrogenesis, SOX, extracellular matrix

## Abstract

**Highlights:**

**What are the main findings?**

- Peripheral blood-derived mesenchymal stromal cells (PB-MSCs) exhibit typical MSC immunophenotypes and demonstrate robust chondrogenic differentiation in vitro, particularly in 3D culture systems.
- Preclinical and early clinical evidence suggest that intra-articular administration of PB-derived cell-based approaches (often combined with hyaluronic acid (HA)) supports cartilage repair, and biomaterial strategies may further enhance cell adhesion and chondrogenesis.

**What are the implications of the main findings?**

- PB-MSCs represent a minimally invasive, clinically attractive cell source for cartilage repair, with potential to broaden access to regenerative approaches.
- Standardisation of PB-MSC isolation, characterisation, and delivery protocols is essential to reduce variability and enable reliable clinical translation.

**Abstract:**

Articular cartilage, a highly specialised and avascular tissue, exhibits limited regenerative potential following trauma or degenerative conditions such as osteoarthritis (OA). Conventional surgical interventions, including microfracture and autologous chondrocyte implantation (ACI), have shown limited long-term efficacy due to donor site morbidity and restricted cell proliferation. In this context, mesenchymal stromal cells (MSCs) have emerged as a promising alternative owing to their multipotency, self-renewal capacity, and low immunogenicity. While bone marrow (BM) remains the traditional source of MSCs, recent studies have reported that peripheral blood-derived mesenchymal stromal cells (PB-MSCs) may possess chondrogenic, osteogenic, and adipogenic potential comparable to that of BM-derived MSCs. PB-MSCs can be harvested through minimally invasive methods, thereby avoiding the complications associated with BM aspiration. Experimental evidence indicates that PB-MSCs exhibit strong cell viability, proliferative potential, and the ability to synthesise cartilage-specific extracellular matrix proteins, such as type II collagen and sulphated glycosaminoglycans, within three-dimensional scaffolds. Immunophenotypically, PB-MSCs express mesenchymal markers including CD29, CD44, CD90, and CD105 while lacking hematopoietic markers CD34 and CD45. Flow cytometry analyses reveal that CD105^+^ populations increase following cryopreservation, highlighting their clinical utility. In contrast to these experimentally defined PB-MSCs, the term peripheral blood stem cells (PBSCs) is used in clinical studies to describe heterogeneous, non-cultured peripheral blood-derived cell preparations, typically enriched in hematopoietic stem and progenitor cells following granulocyte colony-stimulating factor (G-CSF) mobilisation, without full mesenchymal characterisation. In vitro studies confirm successful tri-lineage differentiation, whereas in vivo investigations have demonstrated effective cartilage regeneration using PB-based clinical approaches, including postoperative intra-articular administration of hyaluronic acid (HA) combined with PBSCs, as well as implantation of PBSCs covered with a collagen membrane. Furthermore, advancements in biomaterial engineering, such as poly(ethylene glycol)–cysteine–arginine–glycine–aspartic acid (PEG-CRGD) hydrogels, have enhanced PB-MSC adhesion, proliferation, and chondrogenic differentiation while promoting immunomodulation through M2 macrophage polarisation. Despite these promising outcomes, the available evidence remains limited and heterogeneous, with substantial variability in cell definitions, experimental models, and clinical study designs, which currently constrains definitive conclusions regarding clinical efficacy. Future research should focus on optimising isolation protocols, understanding molecular pathways governing PB-MSC chondrogenesis, and standardising clinical applications. Overall, PB-MSCs represent a viable, less invasive, and translationally relevant cell source for cartilage regeneration and regenerative orthopaedic therapies

## 1. Introduction

Articular hyaline cartilage is a specialised tissue found in weight-bearing, diarthrodial joints. It provides a low-friction surface that absorbs, cushions, and protects the underlying bone from the forces generated during joint movement. However, its lack of blood and lymphatic vessels makes it difficult for the cartilage to regenerate or heal, often severely hindering its ability to repair itself after injury [1,2,3]. Osteoarthritis (OA) and trauma are highly prevalent and economically burdensome, frequently resulting in full-thickness cartilage defects that lack the capacity to heal effectively, leading to long-term disability [4,5,6,7]. When non-surgical treatments prove unsuccessful, current invasive procedures—such as arthroscopic microfracture, autologous chondrocyte implantation (ACI), high tibial osteotomy, or partial knee replacement—typically offer limited success in restoring fully functional cartilage [8,9,10,11].

ACI has been used to treat cartilage defects since its development in 1987 [12,13]. However, numerous published studies from various clinical trial centres have highlighted several inherent limitations of this technique. These include the limited number of cells that can be harvested from the collected tissue, the invasive nature of the initial procedure required to obtain cartilage, and the associated site morbidity. Moreover, it has been reported that autologous cells tend to have limited long-term repair potential due to reduced cell proliferation capacity [14]. As a result, researchers have investigated alternative cell sources, with mesenchymal stromal cells (MSCs) derived from various tissues gaining popularity due to their strong potential for proliferation and differentiation [15,16,17,18].

Over the last two decades, cell-based therapies have advanced to approaches centred on MSCs for cartilage repair, potentially enabling effective single-stage surgical treatments [19]. These mesoderm-derived mesenchymal progenitor cells are a promising source of adult stem cells, as they can differentiate into multiple cell types and exhibit low immunogenicity, making them suitable for widespread use in regenerative medicine [18]. MSCs are fibroblast-like in appearance and are defined by their capacity for self-renewal and differentiation into various mesodermal tissue types, including bone cells (osteoblasts), fat cells (adipocytes), cartilage cells (chondrocytes), and muscle cells (myocytes) [20,21]. Additionally, MSCs have the potential to differentiate into other cell types, such as neurons and astrocytes [21,22].

A growing body of research indicates that peripheral blood (PB) may serve as a viable alternative source of MSCs, demonstrating chondrogenic differentiation capabilities comparable to those of bone marrow-derived MSCs (BM-MSCs) in both laboratory and animal studies [22,23,24]. PB-MSCs can be collected through a minimally invasive method that carries fewer risks compared with BM extraction, which has been linked to complications such as bleeding, long-term pain, nerve and blood vessel damage, and, in rare cases, death [25,26]. PB-MSCs can also be utilised in autologous transplantation, offering significant advantages for patients in clinical settings and supporting the advancement of one-step surgical procedures and other cell-based treatments.

Due to their less invasive collection method and greater availability for clinical use, PB-MSCs have been isolated and utilised as an alternative to BM-MSCs for promoting bone and cartilage regeneration in vivo [26,27]. The literature discussed in this narrative review reflects the current state of research on PB-derived cell populations for cartilage regeneration.

## 2. Synthesis of the Evidence

### 2.1. MSCs of Peripheral Blood Origin

BM has traditionally been considered the primary source of stem cells, and some studies have identified it as the only tissue in the body with a substantial reservoir of readily accessible MSCs [28]. While other sources, like adipose tissue and umbilical cord blood, have also been explored, their availability is limited [29]. In recent years, MSCs have also been identified in PB, though many researchers contend that these cells are present in circulation in only minimal numbers [30,31]. According to Roufosse et al. [32], when tissue injury occurs, MSCs are mobilised from the BM to the site of damage through the PB to aid in regeneration. Based on this concept, researchers have shown that the number of MSCs in the bloodstream can be increased using “blood mobilisation.” This process involves stimulating the BM with granulocyte colony-stimulating factor (G-CSF) to boost cell production [30]. However, this stimulation primarily leads to the release of immature progenitor cells, such as blasts, rather than true multipotent MSCs [33]. Furthermore, studies have shown that this technique does not yield MSCs specifically but instead produces a heterogeneous mixture of MSCs, hematopoietic stem cells, and other immature progenitor cells [34].

Wang S et al. [35] found the following: (a) PB-MSCs demonstrated strong cell viability and the ability to proliferate effectively within a 3D environment. (b) They were capable of differentiating into chondrocyte-like cells and producing cartilage-specific extracellular matrix within 3D scaffolds, while showing minimal expression of hypertrophic markers. (c) When cultured in demineralised cancellous bone (DCB) scaffolds, PB-MSCs showed comparable viability and chondrogenic capacity to BM-MSCs. (d) Although type II collagen (COL2) production by both PB-MSCs and BM-MSCs increased over time, it remained lower than the levels produced by early-passage autologous chondrocytes (ACCs) in DCB scaffolds. It is proposed that MSCs obtained from the PB of healthy individuals possess characteristics and chondrogenic differentiation potential similar to those of MSCs derived from BM. Furthermore, when these MSCs undergo chondrogenic differentiation, they are believed to produce extracellular matrix proteins—especially sulphated glycosaminoglycans (S-GAGs)—at levels comparable to those produced by chondrocytes [23].

### 2.2. Characterisation of PB-MSCs

MSCs have been identified by Fernandez et al. [36] in mobilised PB from breast cancer patients, leading to growing interest in using PB as an alternative to BM for tissue engineering. This is largely due to PB’s less invasive collection method and the ability to obtain larger sample volumes. While some studies have successfully isolated MSCs directly from PB [23,24,37,38,39,40,41,42,43], others have reported the presence of progenitor cells in PB that exhibit MSC-like characteristics [44,45,46,47], including the potential to differentiate into multiple mesenchymal lineages—or, in some cases, only one or two [48,49,50,51]. Several studies have reported successful results using PB as a source of chondrogenic cells for repairing cartilage in both animals and humans [41,52,53,54,55]. In this context, it is important to distinguish experimentally defined PB-MSCs from clinically applied peripheral blood stem cell preparations (PBSCs), which represent heterogeneous, non-cultured PB-derived cell populations, typically enriched in hematopoietic stem and progenitor cells following G-CSF mobilisation and lacking full mesenchymal characterisation.

According to the minimal criteria proposed by the International Society for Cellular Therapy (ISCT) [56,57], mesenchymal stromal cells must fulfill three defining characteristics: plastic adherence under standard culture conditions, tri-lineage differentiation potential, and a specific immunophenotype characterised by expression of CD73, CD90, and CD105 and absence of hematopoietic markers such as CD34, CD45, CD14, CD11b, CD79a, or HLA-DR [58]. Importantly, the ISCT later clarified that the term “mesenchymal stromal cells” should be preferred over “mesenchymal stem cells”, as stemness is rarely demonstrated at a clonal level and most isolated populations represent heterogeneous stromal progenitors rather than true stem cells. This distinction is particularly relevant in peripheral blood-derived cell populations, where several studies report expression of additional adhesion molecules such as CD29 and CD44. Although commonly detected, these markers are not sufficient to define mesenchymal identity and may also be present in activated hematopoietic or progenitor cell fractions. Consequently, many circulating PB-derived populations described in the literature likely represent mesenchymal progenitor or stromal-like cells rather than fully characterised MSCs according to ISCT criteria. For this reason, throughout the present review, the terminology “PB-derived mesenchymal stromal cells” is used cautiously, and studies are interpreted according to the degree of compliance with ISCT minimal criteria.

Blood cell separation is the most widely used technique for collecting PB-MSCs. This method is well-established, straightforward, and commonly used to treat systemic blood disorders. In monocyte suspensions obtained through this process, CD105^+^ cells are found in greater numbers than CD34^+^ cells, and the proportion of CD105^+^ cells has been observed to increase following cryopreservation. In a study by Saw et al. [53], flow cytometry was used to quantify CD34^+^ hematopoietic stem cells (HSCs) and CD105^+^ MSCs. Their findings showed that fresh PB-MSC suspensions contained 7.24% CD105^+^ cells, equivalent to 2.32 × 10^6^ cells/mL [53]. After cryopreservation, this proportion increased to 8.39% (2.69 × 10^6^ cells/mL) [53]. However, the percentage of CD105^+^ cells reported can differ between studies. For example, Turajane et al. [51] found a lower proportion of CD105^+^ cells, ranging from 0.75% to 0.88%. This variation is likely due to differences in patient age: Saw et al. [51] involved younger participants, whereas Turajane et al. [51] involved older participants. They also found that flow cytometry analysis of autologous activated PB-MSCs showed positive expression of mesenchymal surface markers CD29, CD44, CD90, and CD105 [51,53]. Among these, CD29 and CD44 were particularly prominent, with over 80% of the total cell population staining positive for these markers [51,53].

Giovannini et al. [44] demonstrated that mesenchymal progenitor cells (MPCs) derived from PB possess the ability to undergo tri-lineage mesenchymal differentiation, i.e., adipogenic, osteogenic and chondrogenic. In a study by Hopper et al. [41], MSCs were seeded at a density of 1.5 × 10^5^ cells per well in a 12-well plate and cultured as a monolayer for 21 days. Once the cells reached confluency, they were exposed to three differentiation media in triplicate, with basic medium serving as a negative control.

Given the heterogeneity of cell populations described in the literature, clear operational definitions were applied to ensure consistent terminology throughout this review (Table 1).

### 2.3. Tissue-Specific Differentiation

Demonstration of tri-lineage differentiation represents a defining functional property of mesenchymal stromal cells according to ISCT criteria. Across the available literature, peripheral blood-derived cell populations have shown variable differentiation capacity depending on isolation strategy, culture conditions, and degree of phenotypic purification. Successful chondrogenic differentiation of PB-derived stromal populations has been characterised by deposition of sulphated glycosaminoglycans and expression of cartilage-related genes such as SOX9, COL2A1, and aggrecan [23]. In comparative experimental settings, the chondrogenic potential of PB-derived cells was generally lower than that of bone marrow-derived MSCs but comparable to adipose-derived stromal cells when cultured in three-dimensional environments. However, some circulating progenitor populations failed to produce stable cartilage matrix, suggesting incomplete mesenchymal commitment. Osteogenic differentiation has been inconsistently demonstrated. While several studies observed mineralised matrix formation and expression of osteogenic markers, expansion potential and mineralisation capacity were typically inferior to bone marrow-derived MSCs, indicating a more immature or heterogeneous progenitor phenotype [59]. Adipogenic differentiation appears to be the most variable lineage, with some PB-derived populations demonstrating lipid vacuole formation and others failing to differentiate, further supporting the concept that many circulating populations represent mesenchymal progenitors rather than fully established stromal cells [60].

### 2.4. Harvesting and Delivery Methods of PB-MSCs

**Figure 1:**
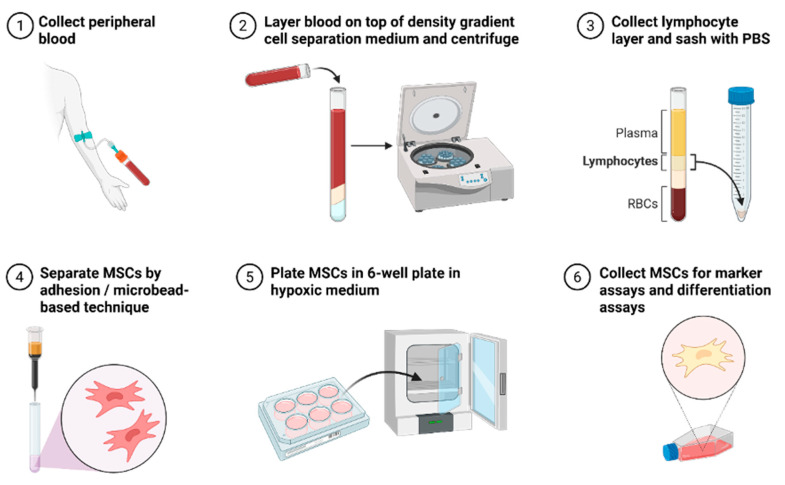
Collection technique to isolate MSCs from PB for clinical application and testing.

Various techniques have been employed to enhance the yield of putative MSCs. Conventional techniques such as density gradient centrifugation and plastic adherence have been shown to effectively isolate MSCs from PB (Figure 1). Kim et al. [61] demonstrated that PB-MSCs closely resemble BM-MSCs in both morphology and immunophenotype. Similarly, Chong et al. [23] successfully isolated MSCs from non-mobilised human PB, finding that these cells displayed tri-lineage differentiation capability and expressed markers such as CD105, CD166, and CD29, while lacking expression of CD34 and CD45. Additionally, the PB-derived MSCs shared comparable features and chondrogenic differentiation capacity with BM-MSCs.

An overview comparing commonly used PB collection strategies, isolation approaches, resulting cell populations, and reported clinical applications is summarised in Table 2.

Kassis et al. [30] successfully isolated a substantial number of human MSCs from PB-MSCs using fibrin microbead-based isolation, following mobilisation of PB in healthy adults with G-CSF. Tondreau et al. [64] reported that human MSCs can be effectively isolated from PB using three approaches: simple plastic adherence, plastic adherence combined with 5% BM-MSC-conditioned medium (CM), and magnetic bead-based selection of CD133-positive cells using an anti-CD133 antibody. The isolated PB-MSCs were verified by their expression of characteristic MSC surface markers and their ability to differentiate into chondrocytes, osteocytes, adipocytes, and even neuronal or glial cell types [64]. Similarly, Hopper et al. [41] extracted PBMCs from healthy donors. After two weeks of culture under hypoxic conditions, the adherent PBMCs developed a fibroblast-like morphology, with 94% of the cells expressing MSC markers, including Stro-1, CD90, CD106, CD105, CD146, CD166, and CD44 [41]. In contrast, only 41% of cells expressed these markers when cultured under normoxic conditions [41].

Certain studies have used pathological conditions to stimulate the release of mesenchymal progenitor cells into the bloodstream. For example, Alm et al. [62] successfully isolated circulating, plastic-adherent MSCs from patients with hip fractures but were unable to do so in women with hip OA. Likewise, Bui K et al. [63] identified MSCs in the blood of 18 out of 58 patients undergoing extracorporeal membrane oxygenation (ECMO). After three passages, over 95% of these cells exhibited typical MSC characteristics and could expand through 3 to 17 population doublings. MSCs were not detected in any of the six healthy control blood samples [63].

Despite various efforts, some studies have failed to detect viable progenitor cells in either normal or mobilised PB. Lazarus et al. [65] were unable to isolate MSCs from peripheral blood progenitor cells (PBPCs) collected via leukapheresis from G-CSF-mobilised donors. Similarly, Wexler et al. [31] assessed the frequency, characteristics, and differentiation potential of MSCs in PB-MSC populations from healthy donors, as well as from adult BM and cord blood (CB). They found that cultured CB- and PB-MSCs did not survive beyond the first passage and produced only sparse, loosely adherent macrophage-like cells with a hematopoietic phenotype (CD45^+^, CD14^+^) [31].

In contrast, a 2008 study by Lund et al. [66] isolated fibroblast-like cells from three of six G-CSF-mobilised PB samples. These cells displayed MSC-like surface markers and could differentiate into adipogenic and osteogenic lineages [66]. However, their chondrogenic potential was minimal, and they showed limited expansion capacity, becoming senescent within 20 to 25 days post-isolation [66]. Additionally, the PB-derived colony-forming cells lacked telomerase activity and exhibited telomere shortening [66]. More recently, Hoogduijn et al. [67] reported that MPCs or MSCs could not be detected in the PB of either healthy individuals or critically ill patients.

Saw et al. [53] documented successful articular cartilage regeneration in five patients who underwent arthroscopic subchondral drilling followed by weekly intra-articular injections of autologous PBPCs combined with hyaluronic acid (HA). The PBPCs were collected by apheresis after G-CSF mobilisation [53]. Flow cytometry analysis revealed that the majority of the collected cells were CD105-positive [53]. On average, 17.6 months after the initial procedure, second-look arthroscopy and tissue biopsies confirmed cartilage regeneration, with histological analysis showing characteristics typical of hyaline cartilage [53].

Skowroński et al. [68] evaluated the long-term clinical outcomes of 52 patients with International Cartilage Repair Society (ICRS) grade III or IV cartilage lesions who were treated using a technique combining microfracture with the transplantation of autologous “blood stem cells,” followed by coverage with a collagen membrane, resembling the ACI approach. The study reported that around 90% of patients experienced favourable outcomes.

Saw et al. [54] carried out a randomised controlled trial (RCT) involving 50 patients aged 18 to 50 with ICRS grade III and IV knee cartilage lesions to further evaluate the impact of PB-MSCs on cartilage regeneration. All patients underwent arthroscopic subchondral drilling and were then divided into two groups of 25: a control group receiving HA alone and an intervention group receiving PB-MSCs combined with HA [52]. Both groups were given five weekly intra-articular injections starting one-week post-surgery [52]. At six months, they received an additional three weekly injections of either HA or PBMSC + HA, depending on the group [54]. At the 18-month follow-up, second-look arthroscopy, biopsy, and MRI assessments in 16 patients from each group showed that the PB-MSC + HA group had superior cartilage repair compared with the HA-only group [54].

A recent case report by Fu et al. [52] described the successful use of PB-MSCs for cartilage repair in a human patient. The MSCs were collected from a 19-year-old male kickboxer who had ICRS grade IV chondral lesions. Treatment involved injecting autologous PB-MSCs beneath a transplanted periosteum flap, along with a patellofemoral realignment procedure. Eight months post-surgery, a second-look arthroscopy revealed a smooth cartilage surface [52]. At a 7.5-year follow-up, the patient had returned to competitive kickboxing [52]. Clinical assessments, including the IKDC 2000 subjective score, Lysholm and Tegner scores, and CT and MRI scans, showed marked improvement compared with preoperative levels [52].

Although the available clinical studies report encouraging outcomes, their findings should be interpreted with caution. Most human investigations are limited by small patient cohorts and non-randomised study designs and frequently rely on case series or uncontrolled comparisons. Clinical outcome assessment often depends on imaging findings or second-look arthroscopy, while histological evaluation is usually restricted to selected patients, limiting generalisability. Variability in cell sources and mobilisation protocols further complicates comparison across studies. Overall, while early clinical data suggest a potential role for peripheral blood-derived cell therapies in cartilage repair, the current level of evidence remains limited and supports the need for adequately powered randomised controlled trials.

### 2.5. Chondrogenicity of PB-MPCs

The extracellular matrix (ECM) of cartilage is composed of several key components, including proteoglycans, HA, COL2, glycoproteins, and a variety of elastic fibres. A majority of the proteoglycans are organised into large aggregates through non-covalent interactions with HA and a link protein. Among these, aggrecan—a large chondroitin sulphate proteoglycan—is especially critical for the proper function of articular cartilage. Together with COL2, aggrecan constitutes a major structural element of cartilage, particularly in joint surfaces. Additionally, aggrecan contributes to essential cell–cell and cell–matrix interactions by binding to HA [69,70,71]. Recent findings indicate that the expression of chondrogenic markers, such as the Sry-type high-mobility group box gene (Sox9) and aggrecan, increases after platelet-rich plasma (PRP) treatment, suggesting that PRP may promote MSC proliferation and support their differentiation into chondrocytes in vitro [72]. Human genetic studies have shown that Sox9 is crucial for determining chondrocyte fate and differentiation. Recognised as the key transcription factor in chondrogenesis, Sox9 regulates a multistep process that activates the expression of target genes such as COL-2 and aggrecan [73].

Four human studies, Kuwana et al. [74], Raghunath et al. [50], Trivanovic et al. [46], and Turajane et al. [51], have investigated the chondrogenic potential of progenitor cells derived from PB. Among them, three reported the successful isolation of chondrogenic MPCs, while Kuwana et al. [74] did not observe chondrogenesis in PB-MPCs from humans. Although these studies demonstrated that circulating, adherent, fibroblast-like cells exhibited chondrogenic or even tri-lineage differentiation potential, they could only be classified as chondrogenic PB-MPCs rather than PB-MSCs. This limitation was due to either incomplete mesenchymal differentiation capacity or the absence of thorough mesenchymal immunophenotyping, leaving the true identity of these progenitor cells uncertain [44,45,46,47,50,51].

Trivanovic et al. [46] discovered fibroblast-like, clonogenic cells in human peripheral blood that demonstrated the ability to differentiate into multiple lineages—osteogenic, chondrogenic, adipogenic, and myogenic. However, the presence of CD34 on approximately 40% of these cells raised doubts about their classification as MSCs, given their atypical immunophenotype [46]. In a separate study, Raghunath et al. [50] isolated MPCs from PBMCs of 10 healthy donors. These cells expressed CD14 and CD105 but not CD34, CD45, or CD133, as confirmed by flow cytometry [46]. The differentiation potential was limited to chondrogenesis, which was successfully demonstrated [46]. Additionally, Turajane et al. [51] used G-CSF-mobilised PBMSCs seeded onto cancellous bone scaffolds for cartilage repair. Their findings showed enhanced expression of cartilage-associated genes, along with increased levels of proteoglycans and glycosaminoglycans [51].

### 2.6. Engineered Chondrogenesis by PB-MSCs

Human mesenchymal stromal cells (hMSCs) offer a promising autologous cell source for repairing large osteoarthritic cartilage defects [75] and can be directed toward chondrogenic differentiation under specific biochemical and mechanical stimuli [76]. Culturing hMSCs in three-dimensional (3D) environments has been shown to enhance their chondrogenic potential [77,78,79,80,81]. Given the critical role of 3D culture conditions, numerous studies have explored the encapsulation of MSCs within various hydrogel scaffolds. These scaffolds are made from both synthetic and natural materials, such as HA [78], polyethylene glycol (PEG) [81], agarose [80], alginate [77], and collagen [79], each with its own set of strengths and limitations. Hydrogels are appealing as cell delivery systems due to their elasticity and high water content, which support extracellular matrix (ECM) development and efficient nutrient exchange, while also physically enclosing cells within their cross-linked structure [77,79]. Their biodegradability is advantageous, as it enables cell movement and the gradual replacement of the scaffold with newly formed tissue. In hydrogels composed of natural materials like collagen and fibrin, degradation is driven by cellular activity. These native substances also contain specific molecular features that promote cell attachment and biological recognition [77].

**Figure 2:**
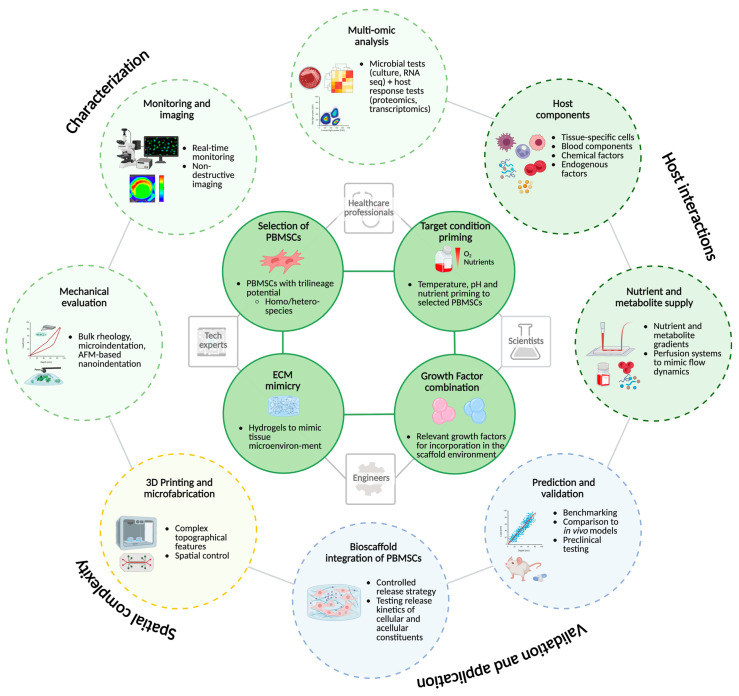
Complex interplay in integrating PBMSCs into hydrogels and development of bioscaffolds for clinical use.

Yang et al. [82] developed a PEG-based hydrogel functionalised with a CRGD peptide and covalently cross-linked via a Michael addition reaction. CRGD enhanced the interaction between PBMSCs and the PEG hydrogel by specifically promoting cell adhesion and proliferation [82]. This hydrogel system was designed to support tissue engineering applications by enhancing immunomodulation and promoting the chondrogenic differentiation of MSCs [82]. Their findings demonstrated that incorporating CRGD improved the interaction between PBMSCs and the PEG hydrogel [82]. Among the tested concentrations, PEG hydrogels modified with 1 mM CRGD showed the greatest enhancement of chondrogenic differentiation [82]. Additionally, CRGD facilitated macrophage polarisation towards the M2 phenotype (an anti-inflammatory role), which is associated with tissue repair and regeneration [82]. These results suggest that PEG-CRGD hydrogels, when combined with PBMSCs, hold promise as scaffold materials for cartilage tissue engineering, as shown in Figure 2.

However, while these biomaterial-based strategies consistently enhance chondrogenic marker expression, extracellular matrix deposition, and immunomodulatory profiles, their translation into constructs with reproducible and clinically relevant mechanical properties has rarely been demonstrated. In particular, most studies do not correlate biomaterial-induced biological outcomes with quantitative biomechanical parameters, limiting conclusions regarding functional cartilage performance. Given the heterogeneity in cell identity and mesenchymal characterisation, the included studies were classified accordingly, as summarised in Table 3.

### 2.7. Future Perspectives

As of 2024–2025, limited recent research provides detailed mechanical properties, such as compressive modulus, tensile modulus, and viscoelastic behaviour, of constructs specifically derived from PB-MSCs. Few studies offer both substantial fold-change quantification of chondrogenic marker expression and corresponding mechanical performance data in PB-MSC-based cartilage constructs. In particular, most PB-MSC studies either omit biomechanical testing entirely or report only surrogate outcomes (e.g., histology or gene expression) without providing quantitative measures of compressive stiffness, tensile strength, viscoelastic behaviour, or fatigue resistance of the regenerated tissue. For clinical translation, PB-MSC-based cartilage constructs will need to demonstrate reproducible structure–function relationships, including mechanical properties approaching those of native articular cartilage, in combination with validated imaging readouts and long-term durability data. Few studies report both quantitative fold-changes in chondrogenic marker expression (e.g., SOX9, COL2, aggrecan) and corresponding functional mechanical performance data within the same PB-MSC-based cartilage constructs. Much of the existing literature instead focuses on PBMCs, cell migration, or gene expression, or uses scaffolds without clearly identifying the MSC source. Moreover, substantial heterogeneity exists across cell populations and methodologies, as many studies do not fully characterise PB-derived cells according to established mesenchymal criteria, limiting comparability across studies and hindering translational interpretation. Standardisation of isolation protocols, multicentre RCTs, objective imaging, and mechanical endpoints are needed to improve the quality of PB-MSCs. Future studies should therefore prioritise standardised PB-MSC isolation and characterisation protocols, multicentre RCT designs, and the integration of objective outcome measures, including quantitative magnetic resonance imaging (MRI) and biomechanical testing, to enable robust structure–function correlations and enhance clinical relevance.

## 3. Conclusions

Peripheral PB-MSCs offer a minimally invasive, clinically viable alternative to BM-MSCs for cartilage regeneration. Preclinical studies consistently demonstrate tri-lineage differentiation, immunomodulatory properties, and the ability to synthesise cartilage-specific extracellular matrix within 3D scaffolds, highlighting their therapeutic promise. Advances in biomaterials, such as PEG-CRGD hydrogels, further enhance their chondrogenic potential and support tissue repair. However, current clinical evidence remains limited by small study cohorts, heterogeneous cell populations, and non-randomised study designs. Accordingly, while PB-MSCs show promise for regenerative orthopaedic applications, further well-designed and adequately powered clinical studies are required. Future research should focus on refining isolation techniques, elucidating molecular mechanisms, and standardising clinical protocols to fully harness their translational potential in cartilage repair.

## Figures and Tables

**Table 1:** Operational definitions of cell populations. (PB-MSCs, peripheral blood-derived mesenchymal stromal cells; PB-MPCs, peripheral blood-derived mesenchymal progenitor cells; PBMCs, peripheral blood mononuclear cells; PBPCs, peripheral blood progenitor cells; BM-MSCs, bone marrow-derived mesenchymal stromal cells; HSCs, hematopoietic stem cells).

Cell Population	Operational Definition
PB-MSCs	Plastic-adherent cells derived from peripheral blood expressing mesenchymal markers (CD29, CD44, CD73, CD90, CD105), lacking hematopoietic markers (CD34, CD45), and demonstrating tri-lineage differentiation capacity.
PB-MPCs	Circulating progenitor cells with partial mesenchymal characteristics and limited or incomplete fulfillment of MSC immunophenotypic or functional criteria.
PBMCs	Heterogeneous mononuclear cell fraction isolated from peripheral blood by density gradient centrifugation, without implying mesenchymal identity.
PBPCs	Mobilised peripheral blood progenitor cell population obtained after G-CSF stimulation, comprising hematopoietic progenitors and variable mesenchymal-like cells.
BM-MSCs	Bone marrow-derived mesenchymal stromal cells fulfilling established mesenchymal criteria, used as a reference comparator.
HSCs	CD34^+^ hematopoietic stem/progenitor cells lacking mesenchymal differentiation capacity.

**Table 2:** PB collection and isolation strategies. (PB: peripheral blood; PB-MSCs: peripheral blood-derived mesenchymal stromal cells; PBSCs: peripheral blood stem cells; PBPCs: peripheral blood progenitor cells; PB-MPCs: peripheral blood-derived mesenchymal progenitor cells; cMPCs: circulating mesenchymal progenitor cells; MSC: mesenchymal stromal cell; G-CSF: granulocyte colony-stimulating factor; HA: hyaluronic acid).

Collection Method	Mobilisation	Isolation Approach	Typical Cell Population	Phenotypic Characteristics	Reported Yield/Efficiency	Clinical Application	Key References
PB	No	Plastic adherence culture	PB-MSCs	CD73^+^, CD90^+^, CD105^+^, CD34^−^, and CD45^−^	Low, inconsistent	Experimental/preclinical studies	Chong et al. [23]; Hopper et al. [41]
PB	G-CSF	Apheresis followed by culture	PBSCs/PBPCs	Mixed population; MSC markers variably expressed	Higher cell numbers; variable MSC fraction	Intra-articular injection ± HA	Saw et al. [53,54]; Turajane et al. [51]
PB	G-CSF	Fibrin microbead-based selection	PB-MSC	MSC-like phenotype (CD73^+^, CD90^+^, CD105^+^)	Low to moderate	Experimental/translational studies	Kassis et al. [30]
PBMC-derived cells	No	Density gradient isolation + culture	PB-MPCs	Partial MSC marker expression	Variable	Preclinical models	Hopper et al. [41]
Pathological mobilisation	No	Plastic adherence culture	cMPCs	MSC-like but inconsistent phenotype	Very rare	Case-based or exploratory studies	Alm et al. [62]; Bui et al. [63]

**Table 3:** Summary of studies investigating peripheral blood-derived cell populations for chondrogenic applications. (PB: peripheral blood; PB-MSCs: peripheral blood-derived mesenchymal stromal cells; BM-MSCs: bone marrow-derived mesenchymal stromal cells; PBSCs: peripheral blood stem cells; G-CSF: granulocyte colony-stimulating factor; MSCs: mesenchymal stromal cells; s-GAG: sulphated glycosaminoglycans; COMP: cartilage oligomeric matrix protein; ISCT: International Society for Cellular Therapy; HLA-DR: human leukocyte antigen–DR). * For in vitro studies, follow-up duration refers to the culture period.

Study	Study Type	Cell Source and Isolation	Phenotypic Markers	Chondrogenic Outcomes	Follow-Up Duration *	Study Quality
Chong et al., 2012 (human) [23]	In vitro	PB mononuclear cells cultured from 2 mL PB (plastic adherence)	CD105^+^, CD166^+^, CD29^+^; CD34^−^, and CD45^−^	Tri-lineage differentiation; s-GAG and cartilage gene (COMP) similar to BM-MSCs	Culture up to passage 2 days and differentiation assays	Controlled lab comparison with BM-MSC; in vitro only.
Fu et al., 2014 (rabbit) [83]	Animal (rabbit model)	Mobilised PB after G-CSF+ AMD3100; adherent MSC culture	Immune phenotype similar to BM-MSCs	In vitro chondrogenesis + in vivo cartilage defect repair equivalent to BM-MSCs	In vivo cartilage evaluation	Good animal model; direct comparison with BM-MSCs.
Fu et al., 2015 (rat mobilised PB) [84]	In vitro + functional	G-CSF-mobilised rat PB-MSCs	CD90, CD44, CD29, CD73, CD105^+^; CD45, CD11b, CD79a, CD34, and HLA-DR^−^	Tri-lineage including chondrogenic differentiation validated	Colony-forming and differentiation assays	Phenotyping as per ISCT criteria; animal cell source.
Kassis et al., 2006 (human mobilised) [30]	In vitro isolation	G-CSF-mobilised human PB; fibrin microbead selection	CD90^+^, CD105^+^; vimentin and fibronectin; CD45^−^, CD34^−^	Osteo/Adipo/Chondrogenic differentiation shown	Primary culture up to 17–18 days	Focus on isolation methodology; low yield noted.
Systematic Reviews, Zhu et al., [24]	Review (in vitro + animal + clinical)	PB-derived MSCs/PBSCs aggregated	N/A	Majority confirm chondrogenic potential in vitro; in vivo repair seen in animal and some human studies	Variable	Reviews highlight overall evidence and limited high-quality clinical data.

## Data Availability

No new data were created or analysed in this study. Data sharing is not applicable.

## Abbreviations

The following abbreviations are used in this manuscript:

ACI	Autologous Chondrocyte Implantation
BM	Bone Marrow
BM-MSCs	Bone Marrow-Derived Mesenchymal Stromal Cells
CB	Cord Blood
CM	Conditioned Medium
CRGD	Cysteine–Arginine–Glycine–Aspartic Acid (peptide)
ECM	Extracellular Matrix
ECMO	Extracorporeal Membrane Oxygenation
G-CSF	Granulocyte Colony-Stimulating Factor
HA	Hyaluronic Acid
HSCs	Hematopoietic Stem Cells
ICRS	International Cartilage Repair Society
MPCs	Mesenchymal Progenitor Cells
MSCs	Mesenchymal Stromal Cells
OA	Osteoarthritis
PB	Peripheral Blood
PBMCs	Peripheral Blood Mononuclear Cells
PB-MSCs	Peripheral Blood-Derived Mesenchymal Stromal Cells
PBPCs	Peripheral Blood Progenitor Cells
PBS	Phosphate-Buffered Saline
PEG	Polyethylene Glycol
PEG-CRGD	Polyethylene Glycol-CRGD Hydrogel
3D	Three-Dimensional
